# Supplementary material for: Drug Resistance Prediction Using Deep Learning Techniques on HIV-1 Sequence Data
Source: Viruses. 2020 May 19;12(5):560. doi: 10.3390/v12050560 (PMC7290575; doi:10.3390/v12050560)
Supplement: Supplementary file 1 [file viruses-12-00560-s001.pdf]

Supplemental Data

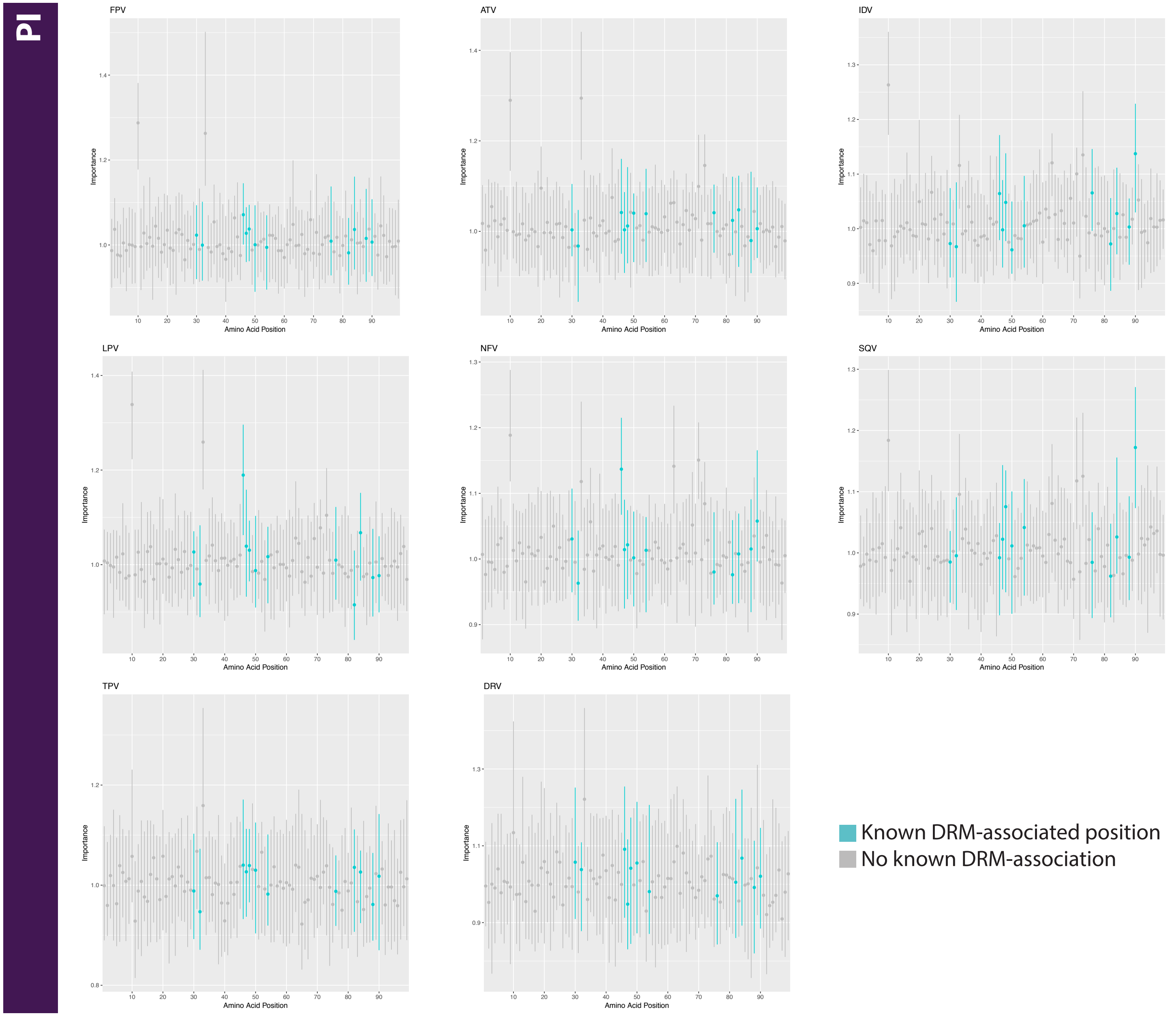

Figure 1. Annotated feature importance plots for the MLP classifier for all features across the whole gene - PI. Horizontal axes are amino acid positions ordered 1-99; vertical axes are feature importance (measured as change in 1-AUC).

Abbreviations: FPV=fosamprenavir; ATV=atazanavir; IDV=indinavir; LPV=lopinavir; NFV=nelfinavir; SQV=saquinavir; TPV=tipranavir; DRV=darunavir; PI=protease inhibitor.

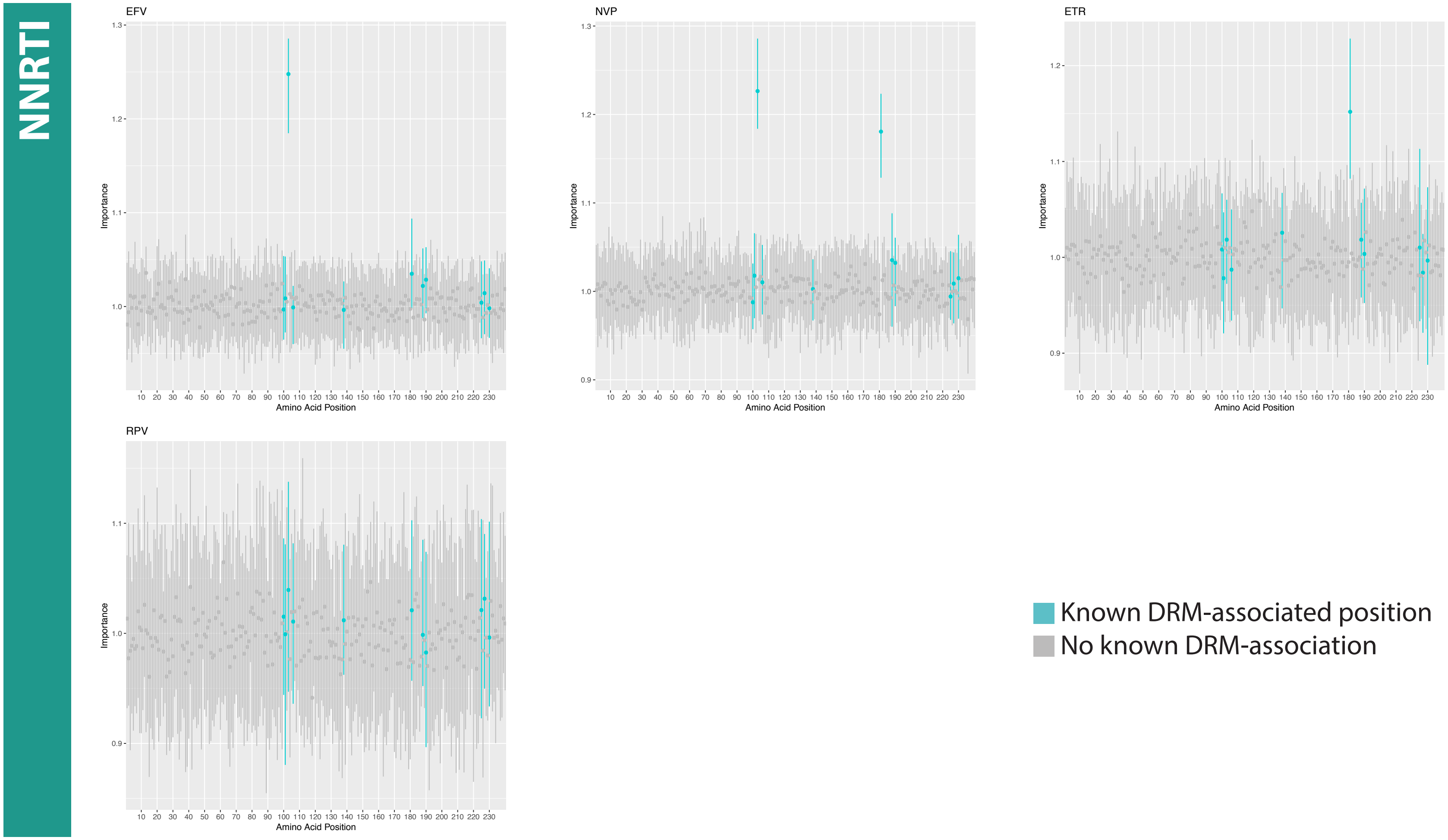

Figure 2. Annotated feature importance plots for the MLP classifier for all features across the whole gene - NNRTI. Horizontal axes are amino acid positions ordered 1-240; vertical axes are feature importance (measured as change in 1-AUC).

Abbreviations: EFV=efavirenz; NVP=nevirapine; ETR=etravirine; RPV=rilpivirine; NNRTI=non-nucleotide reverse transcriptase inhibitor.

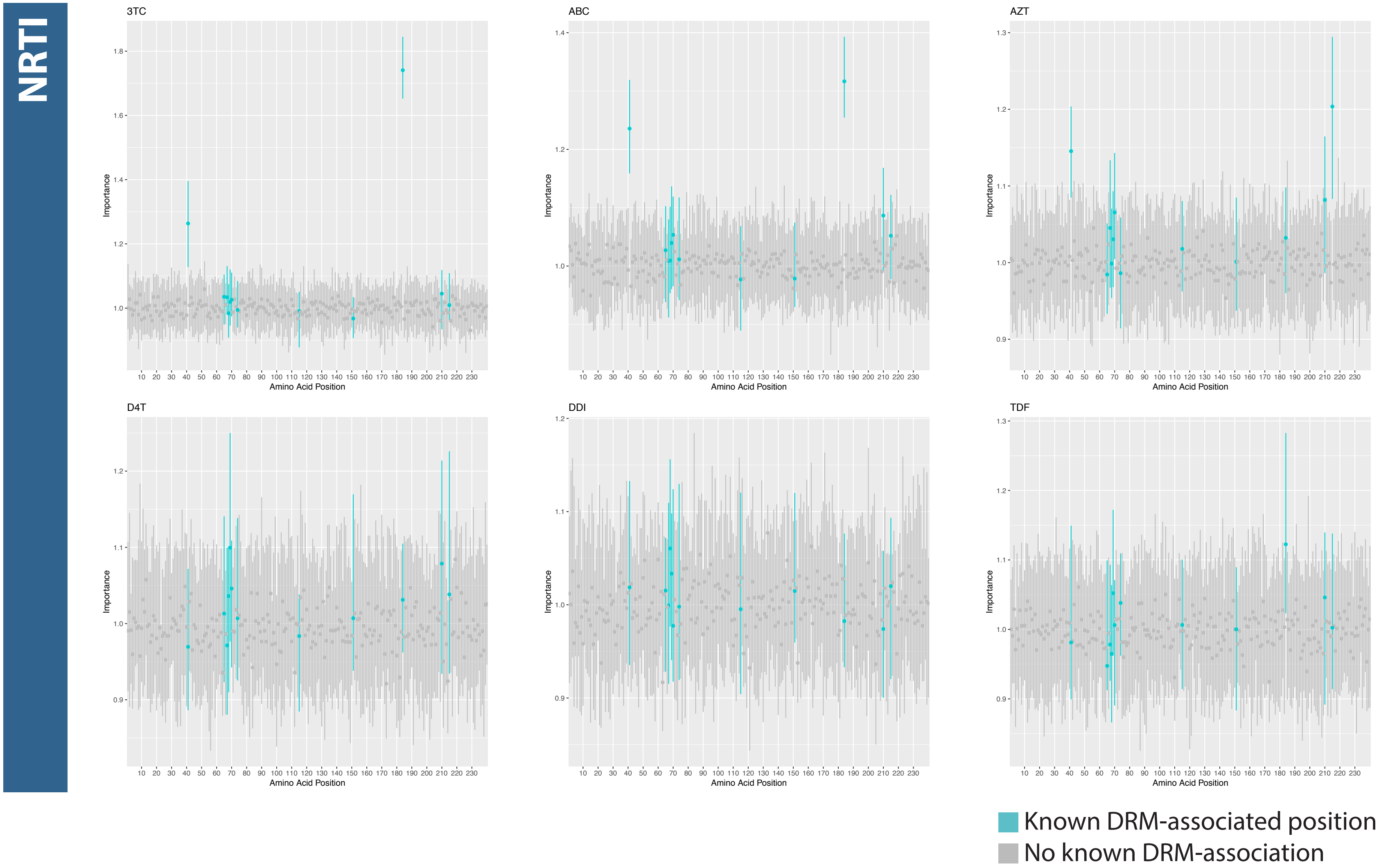

Figure 3. Annotated feature importance plots for the MLP classifier for all features across the whole gene - NRTI. Horizontal axes are amino acid positions ordered 1-99; vertical axes are feature importance (measured as change in 1-AUC).

Abbreviations: 3TC=lamivudine; ABC=abacavir; AZT=azidothymidine; D4T=stavudine; DDI=didanosine; TDF=tenofovir disoproxil fumarate; NRTI=nucleotide reverse transcriptase inhibitor.

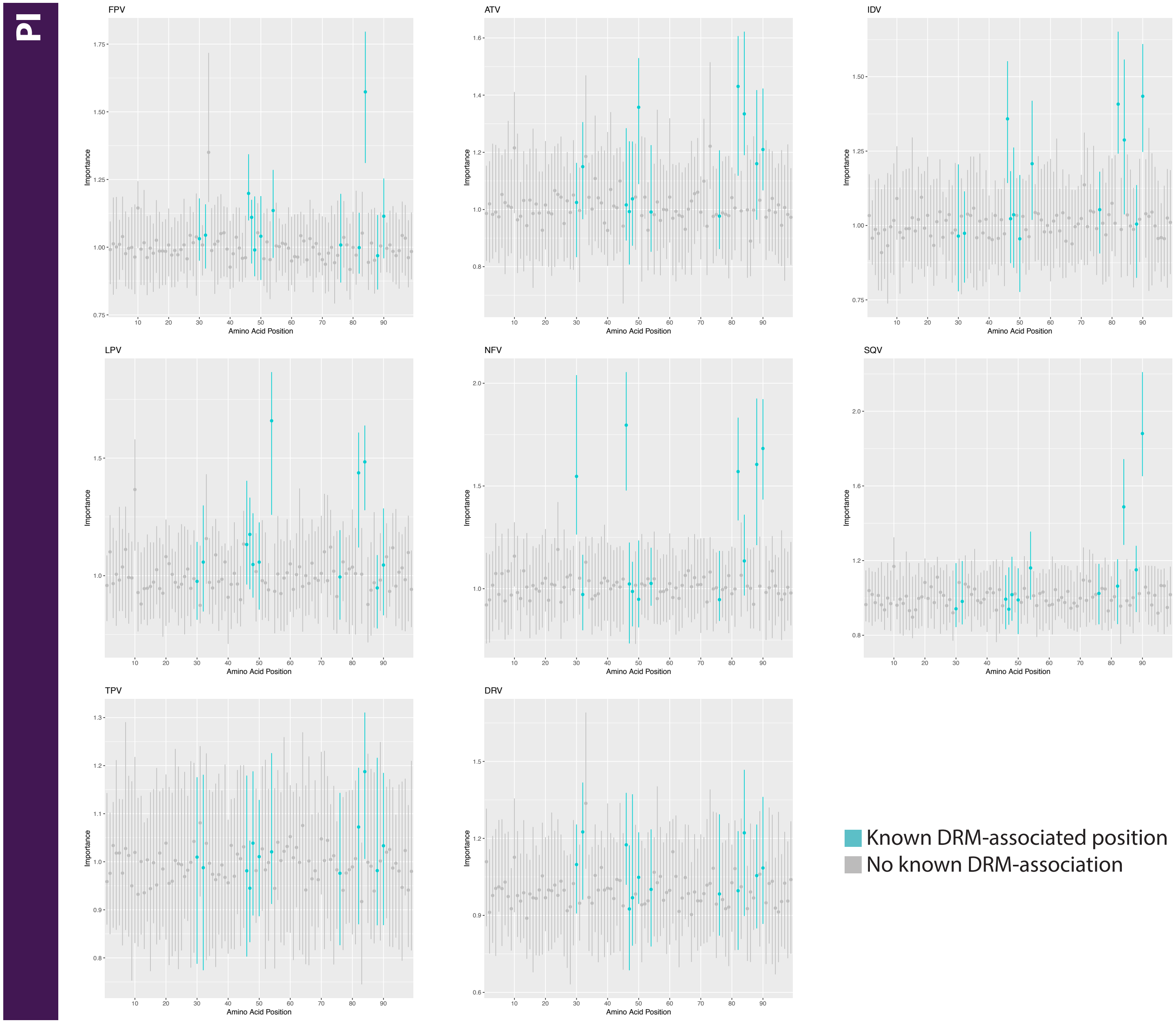

Figure 4. Annotated feature importance plots for the BRNN classifier for all features across the whole gene - PI. Horizontal axes are amino acid positions ordered 1-99; vertical axes are feature importance (measured as change in 1-AUC).

Abbreviations: FPV=fosamprenavir; ATV=atazanavir; IDV=indinavir; LPV=lopinavir; NFV=nelfinavir; SQV=saquinavir; TPV=tipranavir; DRV=darunavir; PI=protease inhibitor.

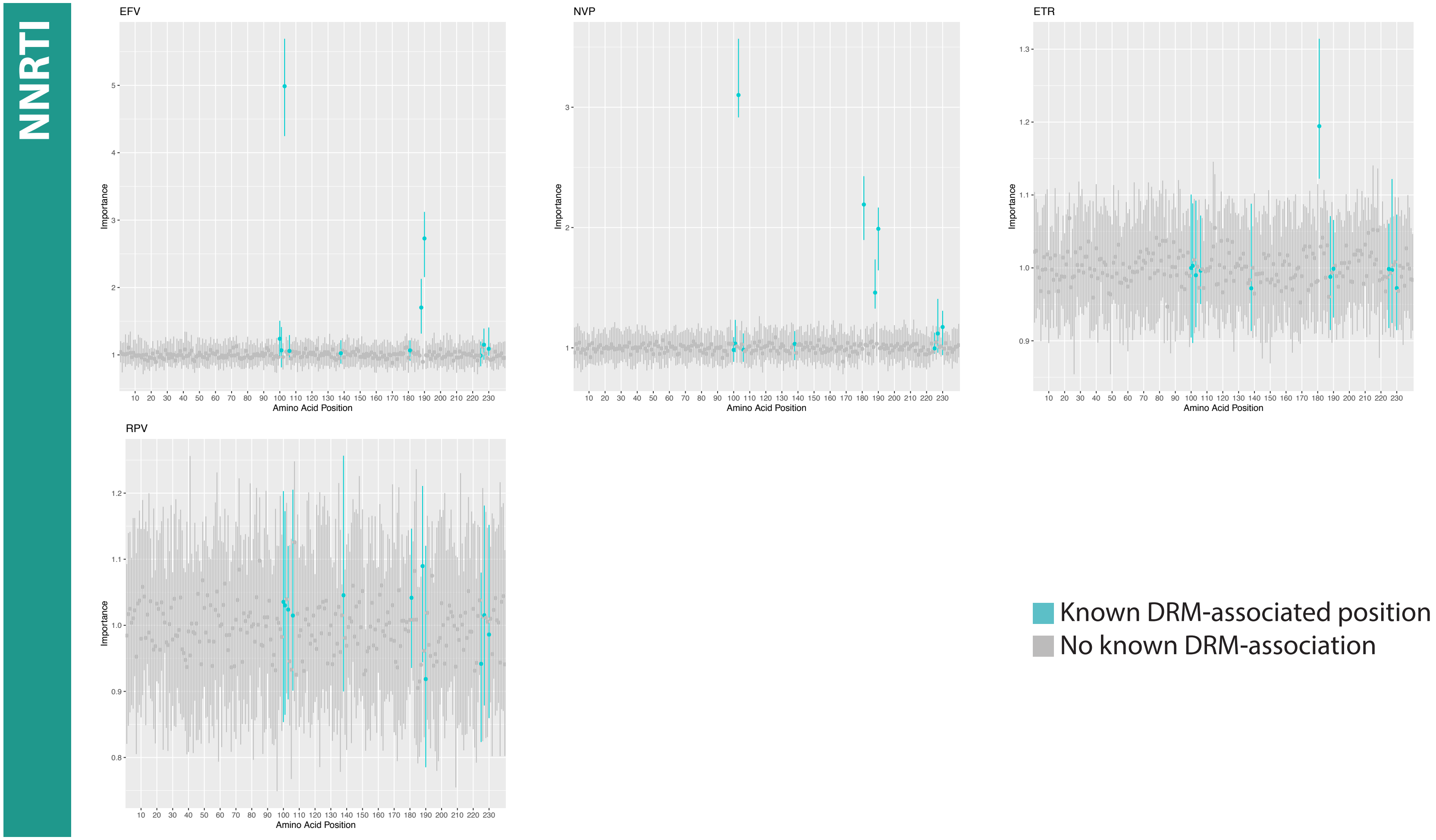

Figure 5. Annotated feature importance plots for the BRNN classifier for all features across the whole gene - NNRTI. Horizontal axes are amino acid positions ordered 1-240; vertical axes are feature importance (measured as change in 1-AUC).

Abbreviations: EFV=efavirenz; NVP=nevirapine; ETR=etravirine; RPV=rilpivirine; NNRTI=non-nucleotide reverse transcriptase inhibitor.

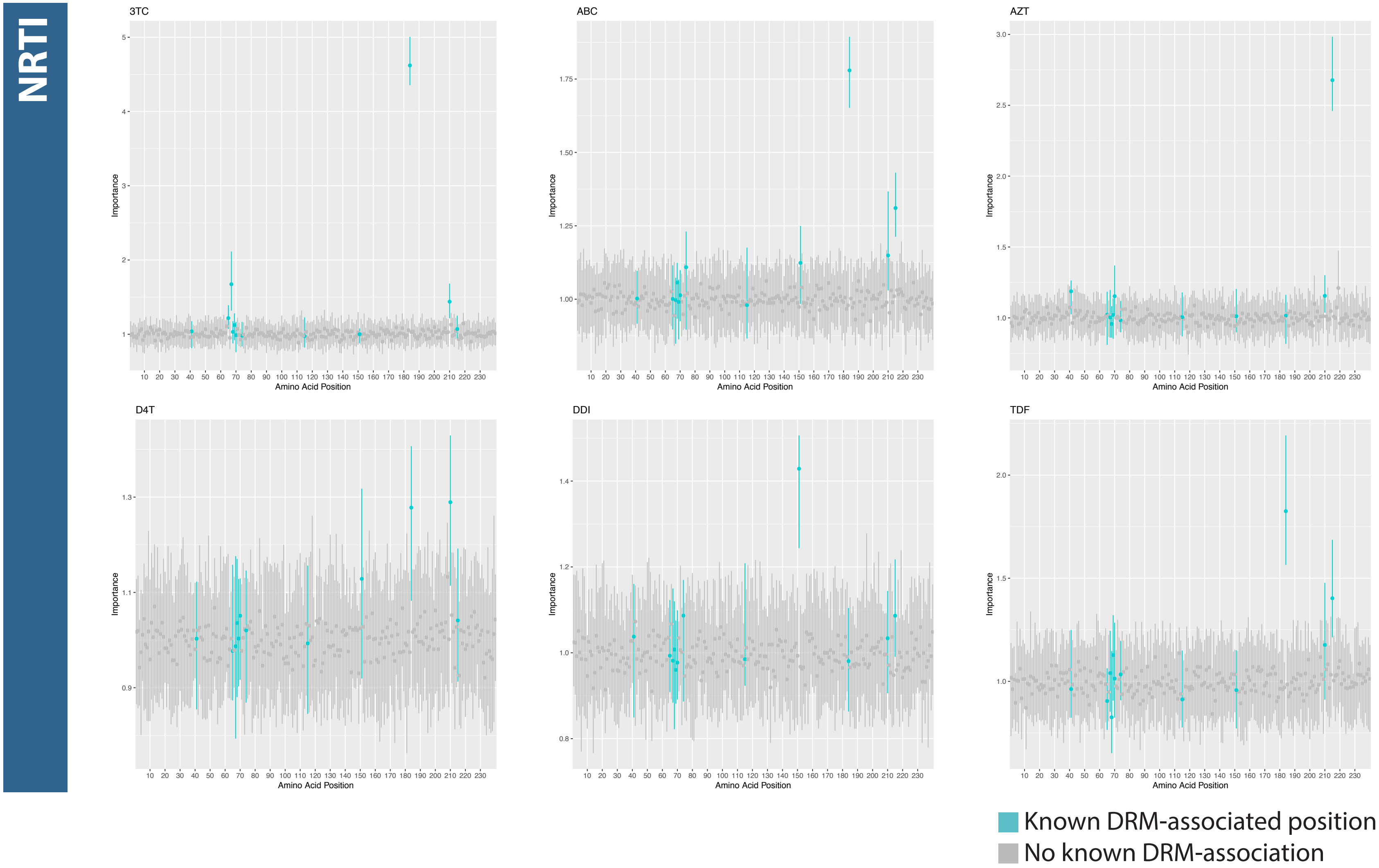

Figure 6. Annotated feature importance plots for the BRNN classifier for all features across the whole gene - NRTI. Horizontal axes are amino acid positions ordered 1-240; vertical axes are feature importance (measured as change in 1-AUC).

Abbreviations: 3TC=lamivudine; ABC=abacavir; AZT=azidothymidine; D4T=stavudine; DDI=didanosine; TDF=tenofovir disoproxil fumarate; NRTI=nucleotide reverse transcriptase inhibitor.

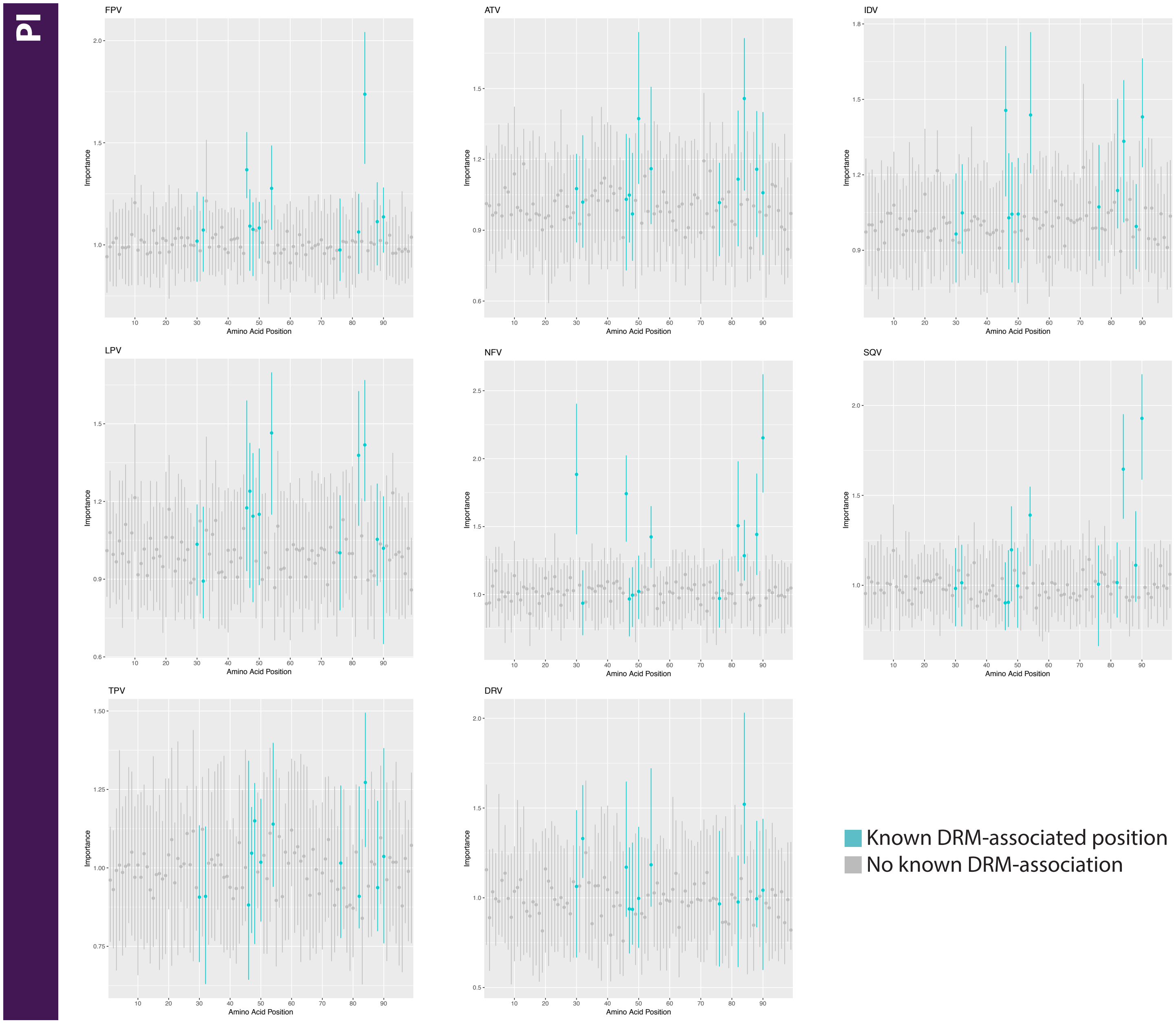

Figure 7. Annotated feature importance plots for the CNN classifier for all features across the whole gene - PI. Horizontal axes are amino acid positions ordered 1-99; vertical axes are feature importance (measured as change in 1-AUC).

Abbreviations: FPV=fosamprenavir; ATV=atazanavir; IDV=indinavir; LPV=lopinavir; NFV=nelfinavir; SQV=saquinavir; TPV=tipranavir; DRV=darunavir; PI=protease inhibitor.

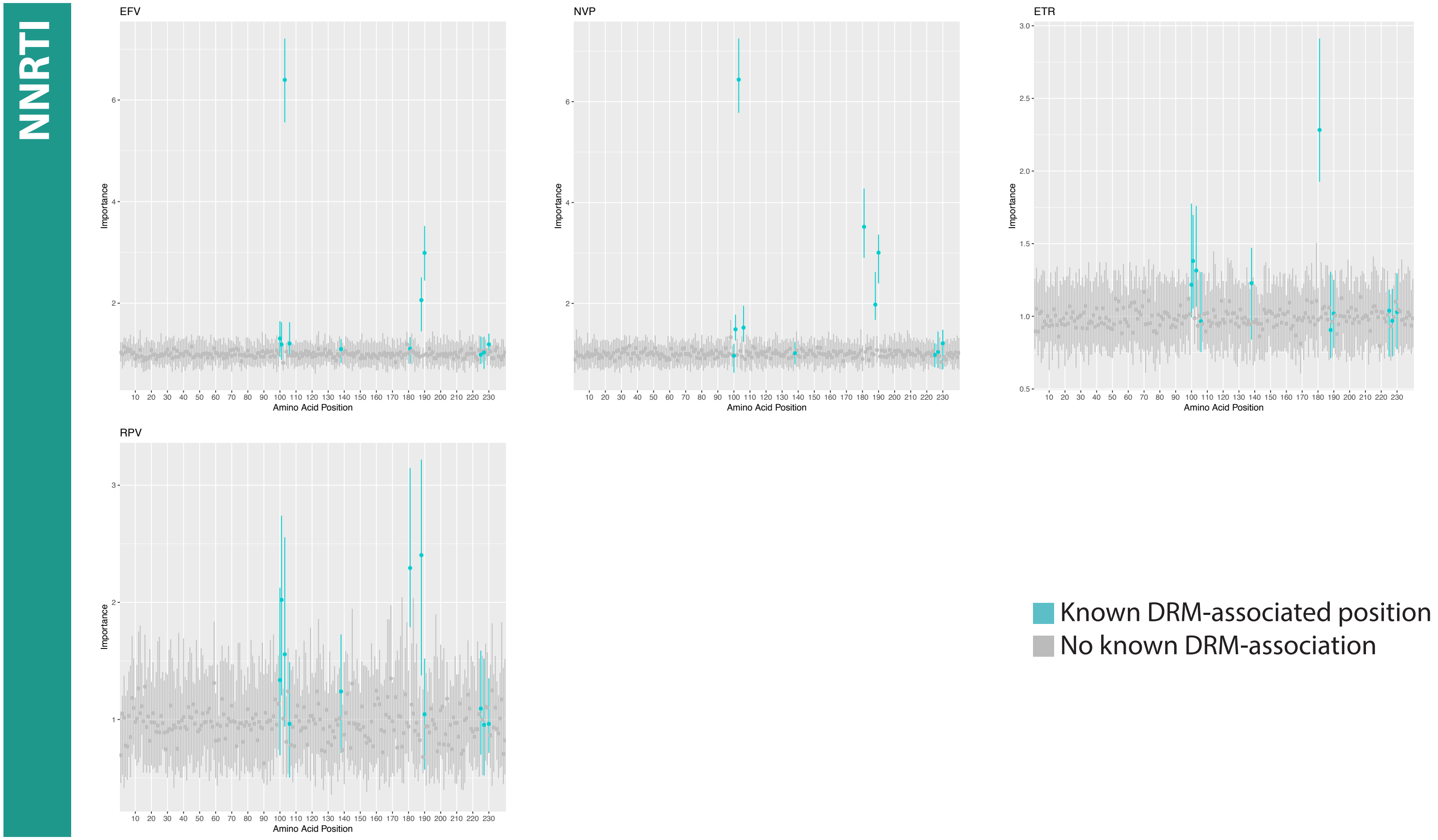

Figure 8. Annotated feature importance plots for the CNN classifier for all features across the whole gene - NNRTI. Horizontal axes are amino acid positions ordered 1-240; vertical axes are feature importance (measured as change in 1-AUC).
Abbreviations: EFV=efavirenz; NVP=nevirapine; ETR=etravirine; RPV=rilpivirine; NNRTI=non-nucleotide reverse transcriptase inhibitor.

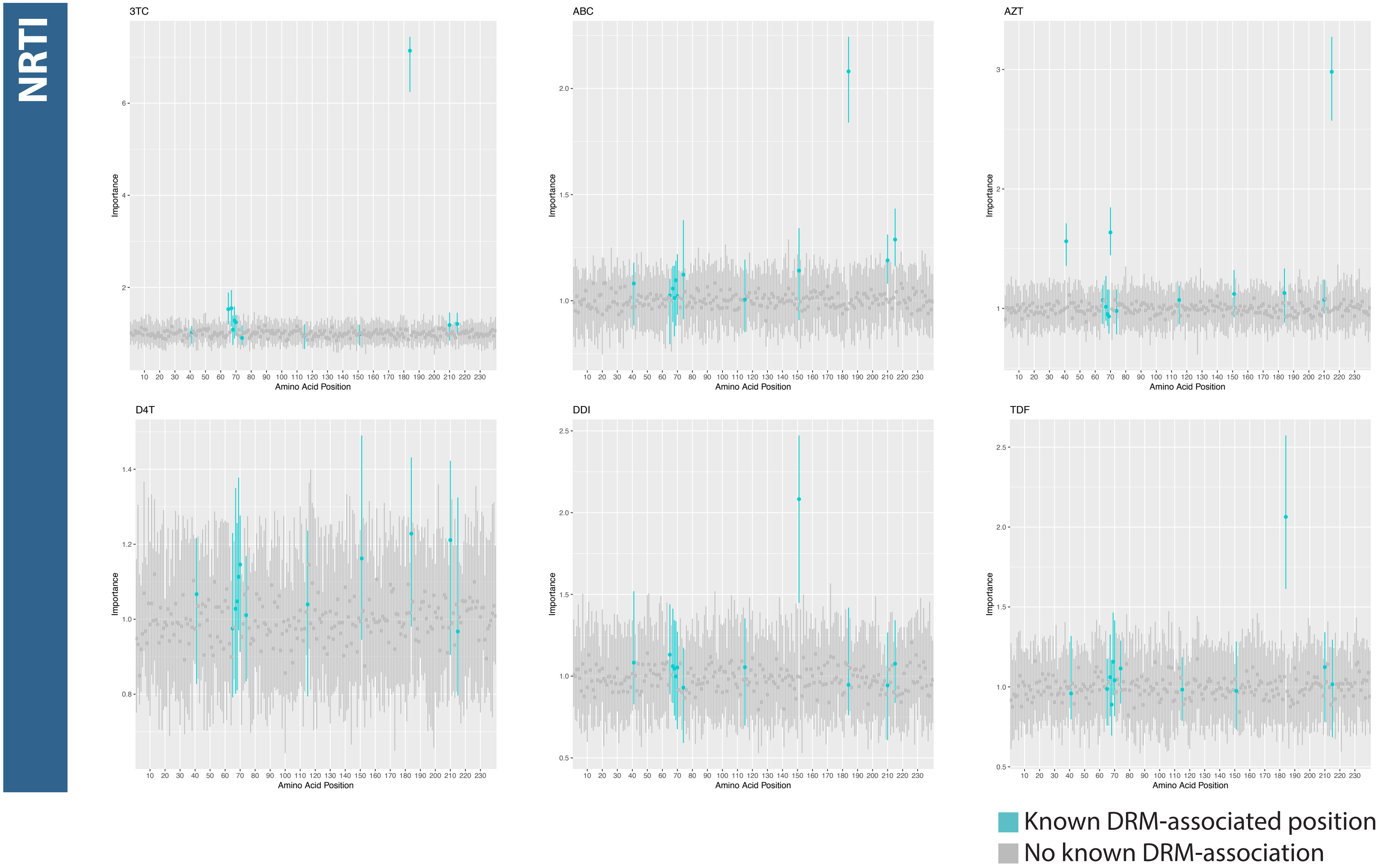

Figure 9. Annotated feature importance plots for the CNN classifier for all features across the whole gene - NRTI. Horizontal axes are amino acid positions ordered 1-240; vertical axes are feature importance (measured as change in 1-AUC).
Abbreviations: 3TC=lamivudine; ABC=abacavir; AZT=azidothymidine; D4T=stavudine; DDI=didanosine; TDF=tenofovir disoproxil fumarate; NRTI=nucleotide reverse transcriptase inhibitor.

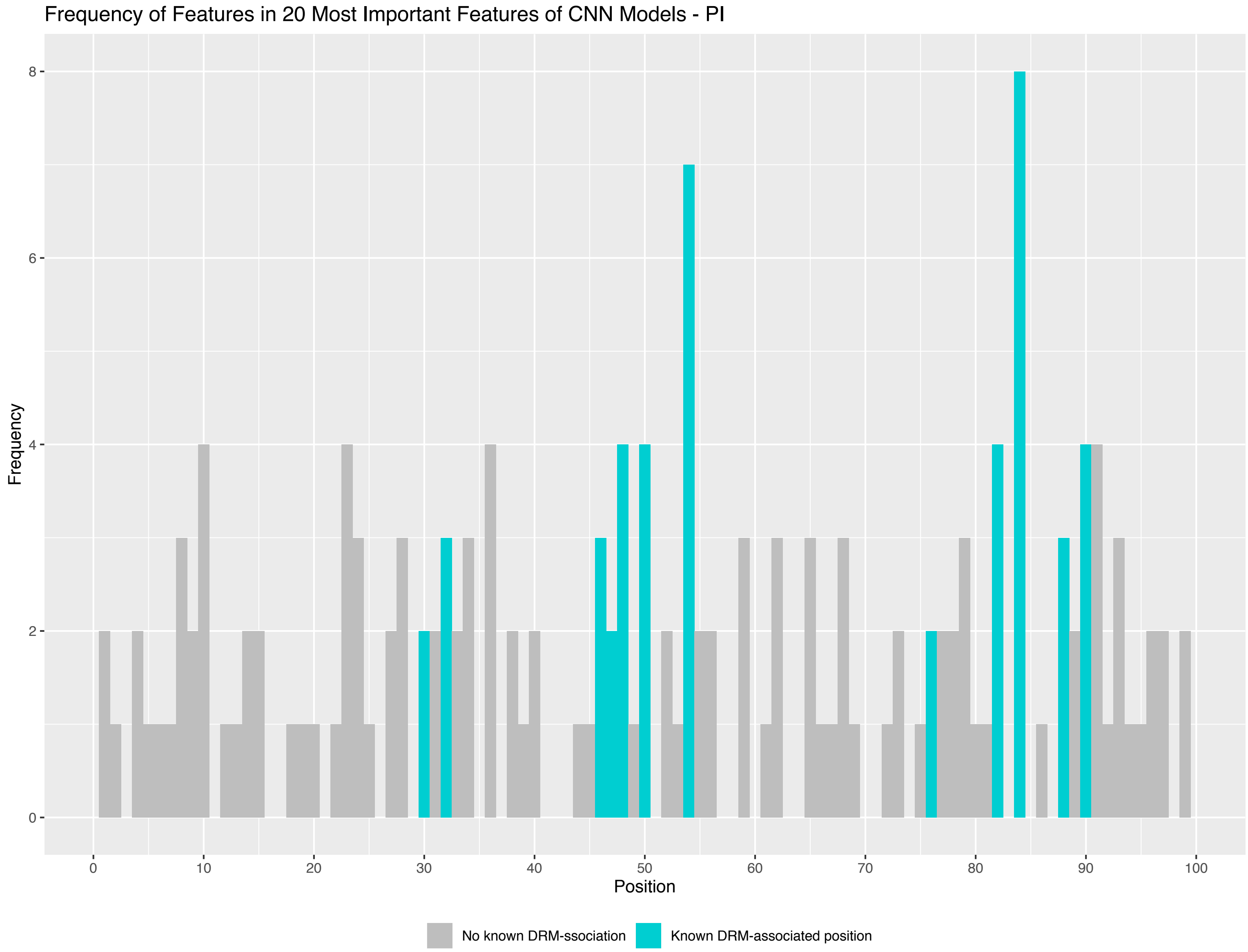

Figure 10. Histogram denoting the frequency of amino acid positions among the 20 most important features for CNN models in PI datasets.
Abbreviations: CNN=convolutional neural network, PI=protease inhibitor, DRM=drug resistance mutation.

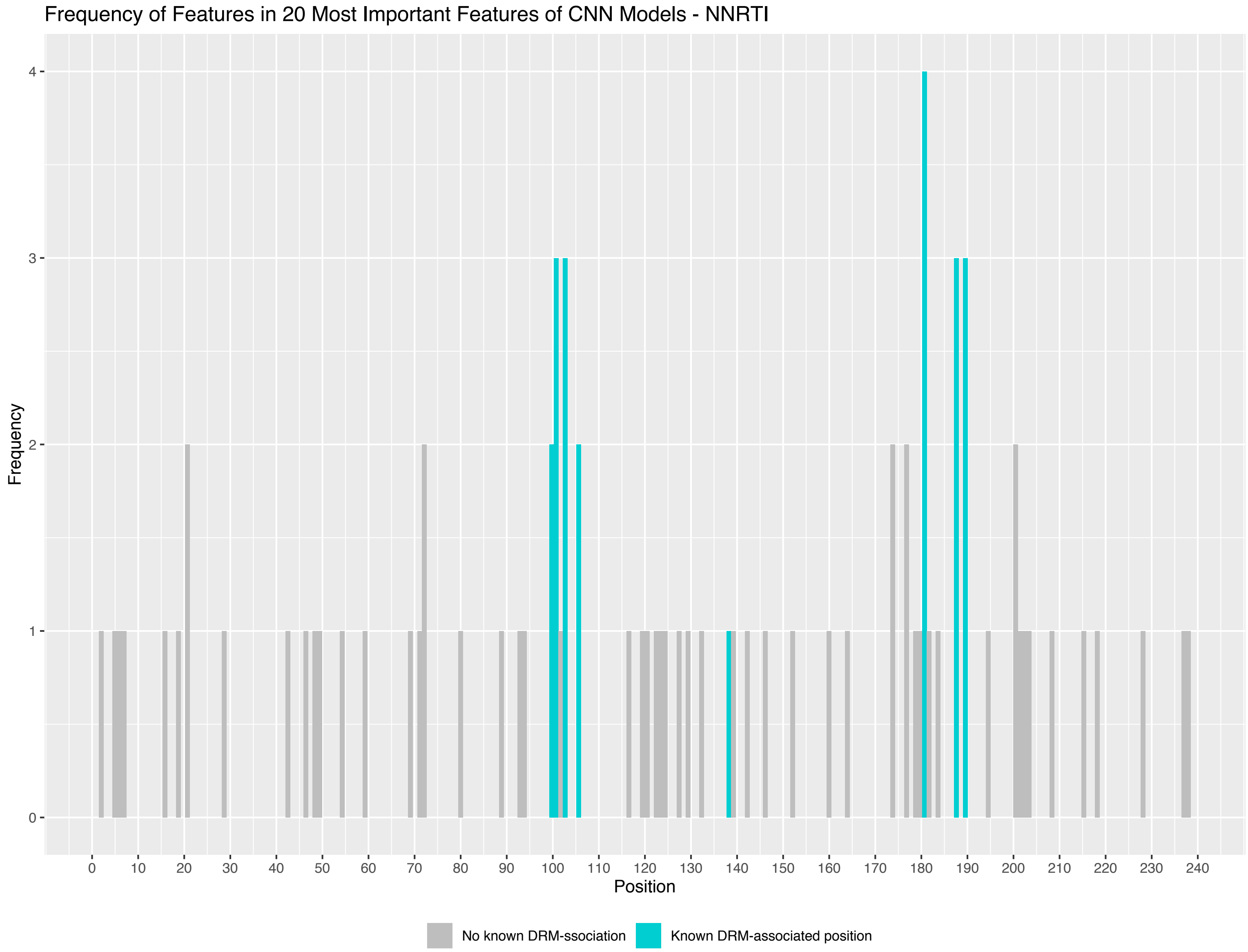

Figure 11. Histogram denoting the frequency of amino acid positions among the 20 most important features for CNN models in NNRTI datasets. *Abbreviations: CNN=convolutional neural network, NNRTI=non-nucleotide reverse transcriptase inhibitor, DRM=drug resistance mutation.*

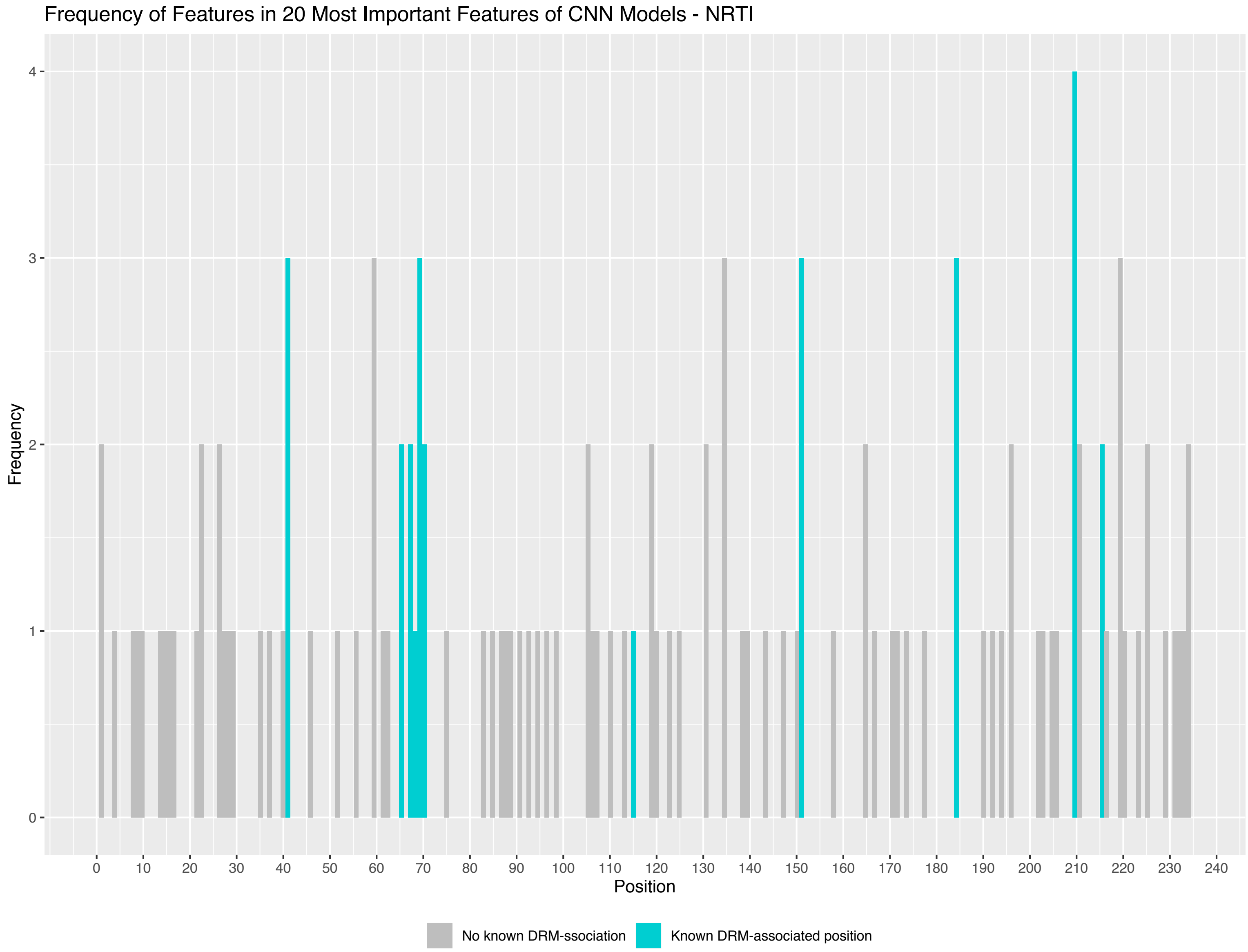

Figure 12. Histogram denoting the frequency of amino acid positions among the 20 most important features for CNN models in NRTI datasets.
Abbreviations: CNN=convolutional neural network, NRTI=nucleotide reverse transcriptase inhibitor, DRM=drug resistance mutation.
